# Assessment of Atrial Fibrillation and Vulnerability in Patients with Wolff-Parkinson-White Syndrome Using Two-Dimensional Speckle Tracking Echocardiography

**DOI:** 10.1371/journal.pone.0108315

**Published:** 2014-11-14

**Authors:** Jing-Jie Li, Fang Wei, Ju-Gang Chen, Yan-Wei Yu, Hong-Yue Gu, Rui Jiang, Xiu-Li Wu, Qian Sun

**Affiliations:** 1 Department of Cardiology, the First Affiliated Hospital of Harbin Medical University, Harbin, P.R. China; 2 Departments of Cardiology, General Hospital of Taiyuan Iron and Steel Company, Taiyuan, P.R. China; University of Minnesota, United States of America

## Abstract

**Purpose:**

The aim was to assess atrial fibrillation (AF) and vulnerability in Wolff-Parkinson-White (WPW) syndrome patients using two-dimensional speckle tracking echocardiography (2D-STE).

**Methods:**

All patients were examined via transthoracic echocardiography and 2D-STE in order to assess atrial function 7 days before and 10 days after RF catheter ablation. A postoperative 3-month follow-up was performed via outpatient visit or telephone calls.

**Results:**

Results showed significant differences in both body mass index (BMI) and supraventricular tachycardia (SVT) duration between WPW patients and DAVNP patients (both *P*<0.05). Echocardiography revealed that the maximum left atrial volume (LAV_max_) and the left ventricular mass index (LVMI) in diastole increased noticeably in patients with WPW compared to patients with DAVNP both before and after ablation (all *P*<0.05). Before ablation, there were obvious differences in the levels of SRs, SRe, and SRa from the 4-chamber view (LA) in the WPW patients group compared with patients in the DAVNP group (all *P*<0.05). In the AF group, there were significant differences in the levels of systolic strain rate (SRs), early diastolic strain rate (SRe), and late diastolic strain rate (SRa) from the 4-chamber view (LA) both before and after ablation (all *P*<0.05). In the non-AF group, there were decreased SRe levels from the 4-chamber view (LA/RA) pre-ablation compared to post-ablation (all *P*<0.05).

**Conclusion:**

Our findings provide convincing evidence that WPW syndrome may result in increased atrial vulnerability and contribute to the development of AF. Further, RF catheter ablation of AAV pathway can potentially improve atrial function in WPW syndrome patients. Two-dimensional speckle tracking echocardiography imaging in WPW patients would be necessary in the evaluation and improvement of the overall function of RF catheter ablation in a long-term follow-up period.

## Introduction

Auricular fibrillation (AF) is an extremely common cardiac arrhythmia widely seen in patients with manifest accessory pathways (AP), which typically take the form of irregular atrial activation and contraction [1], [2]. For those under the age of 65, the annual incidence of AF is estimated at 1.9 per 1000 women and 3.1 per 1000 men each year; however, the incidence each year exceeds 32 patients per 1000 persons 80 years or older, showing that AF prevalence is substantial in the older population which could lead to considerable disability and mortality worldwide [3], [4]. Over the past decades, evidence from epidemiological studies has shown AF to stem from a complicated interaction of hereditary, race, multi-environmental, and genetic factors [5], [6]. A wide variety of risk factors have been identified in the development of AF, such as advancing age, cardiovascular disease, and body mass index. [7]. Furthermore, the existence of a widespread heritable component underlying AF has triggered numerous investigations regarding susceptibility genes and the polymorphisms that predispose AF [8], [9]. Several possible mechanisms responsible for the genesis of AF have been hypothesized, and may provide a better understanding of the complex pathophysiology of AF [10]–[12]. For instance, ectopic foci in pulmonary veins have been recognized as AF triggers, since the focal activities not only induce AF, but they have also been shown to play an important role in sustaining re-entry [13]. Recently, extensive studies have revealed a growing body of evidence indicating that Wolff-Parkinson-White syndrome (WPW) might also be regarded as an important risk modifier influencing AF susceptibility [14], [15].

WPW is one of the most common congenital cardiac abnormalities among the ventricular pre-excitation syndromes, with a prevalence of 0.9% to 3% in the general population [16]. In general, WPW is characterized by the presence of an abnormal accessory electrical conduction pathway which may produce ventricular pre-excitation and paroxysmal reentrant tachycardia [17]. In particular, frequent tachycardias may also promote electrical remodeling and an increased atrial vulnerability to AF, which has been shown to more frequently induce sustained episodes of AF [18], [19]. To date, approximately one third of WPW patients develop AF, which is a fairly high frequency, and accumulating evidence suggests that this can be life-threatening when associated with high ventricular rates stemming from accessory pathway conduction [17], [20]. To be more specific, the presence of AP itself may affect myocardial electrophysiology and contractile properties, thereby increasing AF vulnerability, which is in turn suspected to be involved in the occurrence of AF in patients with the WPW syndrome [21]. Importantly, it should be noted that WPW patients with AF appear to have both reversible and intrinsic AP-related atrial vulnerability, the appearance of which is generally considered to be one of the most important requirements for the development of AF [22]. In addition, the occurrence of AF is closely associated with atrioventricular reentrant tachycardia, the most common form of SVT; this may give rise to acute atrial dilatation, resulting in conduction slowing and a significant increase in AF vulnerability [23], [24]. Due to the complicated pathogenic factors involved in the occurrence of AF in patients with WPW syndrome, the precise mechanism leading to the development of AF in WPW patients is still not entirely understood [21], [24]. It is therefore very important to determine all possible mechanisms for the development of AF in the WPW patients, and a detailed examination before and after radiofrequency (RF) catheter ablation in the present study assists in providing insight into the AF genesis mechanism in patients with WPW syndrome.

## Methods

### Ethics statement

This study was approved by the Ethics Committee of the First Affiliated Hospital of Harbin Medical University. Informed consent was obtained in written form from all participants using procedures approved by institutional review boards. Written consent from the next of kin, caretakers, or guardians was obtained on behalf of all minors/children.

### Study design and subjects

Between April 2012 and December 2012, 28 patients diagnosed with WPW were admitted to the Department of Cardiology in the First Affiliated Hospital of Harbin Medical University. All of these patients were treated via RF catheter ablation of the AAV pathway. In addition, 24 patients with dual atrioventricular nodal pathways (DAVNP) who were treated with RF catheter ablation of the slow pathway were selected as the control subjects. WPW patients exhibiting atrial flutter or atrial fibrillation had ventricular rates of more than 200 beats/min. It should be noted that anti-arrhythmic drugs regularly contribute to the occurrence of the arrhythmogenic effect, which means that these drugs can cause a new occurrence of arrhythmias or aggravate existing arrhythmias. Such effects include increased seizure frequencies of premature ventricular contractions and acceleration of ventricular tachycardia rates; with this in mind, patients exhibiting these symptoms were not suitable for the administration of anti-arrhythmic drugs.

Patients who met any of the following criteria were enrolled in this study: (1) be in accordance with the standardized criteria for echocardiography diagnosis of WPW [25]; (2) exhibit recessive bypass or DAVNP according to transesophageal atrial pacing and intercardiac electrophysiology detection; (3) produce ultrasound images of ideal quality. Patients were excluded if they had: (1) administration of antiarrhythmic drugs within three months prior to admission; (2) cardiac arrhythmia (except for WPW or DAVNP) and conduction disturbances; (3) a history of organic heart disease, chronic pulmonary disease, thyroid disease, or other systemic diseases.

### ECG assessment

All patients were examined via transthoracic echocardiography and two-dimensional speckle tracking echocardiography (2D-STE) to assess atrial function before and after RF catheter ablation. Doppler studies were performed on all patients using a Philips iE33 echocardiograph (Philips Ultrasound) equipped with an S5-1 transducer (2.0∼3.5 MHz). The analysis was performed offline using the EchoPAC program with custom 2D strain rate imaging software. All ECG images were reviewed by a single operator, who was blind to the patients at the time of ECG assessment. ECG parameters were prospectively collected according to current guidelines [26]. The left atrial diameter (LAD), left ventricular end diastolic diameter (LVEDD), left ventricular ejection fraction (LVEF), left ventricular mass index in diastole (LVMI), left ventricular mass index in systole (LV mass s index), left atrial maximum volume (LAV_max_), left atrial minimal volume (LAV_min_), right atrial maximum volume (RAV_max_), right atrial minimal volume (RAV_min_), and left ventricular mass index (LVMI) of each patient were measured and calculated. The strain rate (SR) curve was acquired in the apical four-chamber, two-chamber view, and three-chamber views with 2D-STE. The apical four-chamber view permits two-dimensional SR measurements of both atria. The average peak systolic strain rate (SRs), early diastolic strain rate (SRe), and late diastolic strain rate (SRa) in each segment was measured.

### Follow up

ECG examination was conducted over the 7 days before and 10 days after the RF catheter ablation; the ablation end point was reached when: (1) forward (antidromic) conduction along accessory pathways was blocked; (2) pre-shock disappeared; (3) atrioventricular nodal reentrant tachycardia could not be induced. A postoperative 3-month follow-up was performed with outpatient visit or telephone calls.

### Statistical analysis

All statistical analyses were performed using SPSS 18.0 software (SPSS, Inc., Chicago, IL, USA). Data were presented as mean ± standard deviation (mean ± SD). One-way analysis of variance (ANOVA) and the Student's t-test were used for normally distributed variables, while the Mann-Whitney's U-test was used for non-normally distributed variables. The enumeration data were compared with the chi-square test. Paired sample t tests were applied to the comparisons between groups (before and after ablation) for nominal variables. A probability value of less than 0.05 was considered statistically significant.

## Results

### Baseline characteristics between the experimental group and the control group

Included patients were classified into an experimental group and a control group. The experimental group consisted of 28 WPW patients (17 males and 11 females), with a mean age of 40.0±14.7 years (range, 15∼64 years). The control group included 24 DAVNP patients (11 males and 13 females), with a mean age of 33.0±13.1 years (range, 16∼69 years). Baseline characteristics and ECG results of WPW patients and DAVNP patients are shown in Table 1. There were significant differences in body mass index (BMI) and duration of SVT between WPW patients and DAVNP patients (BMI: 23.3±4.0 vs. 21.0±2.3, *P* = 0.012; duration of SV_male/female_: 7.1±7.7/4.0±8.8 vs. 3.3±4.7/2.0±4.3, *P* = 0.023), but no significant difference was found in any other clinical characteristics, such as sex, age, heart rate, *P* wave duration, etc. (all *P*>0.05).

**Table 1:** Baseline characteristics of WPW patients and DAVNP patients.

	WPW patients (n = 28)	DAVNP patients (n = 24)	*P* value
Age (years)	40.0±14.7	33.0±13.1	0.060
Sex (male/female)	17/11	11/13	0.290
Body surface area (m^2^)	1.7±0.2	1.6±0.2	0.230
BMI (kg/m^2^)	23.3±4.0	21.0±2.3	0.012
Duration of SVT (years)	7.1±7.7/4.0±8.8	3.3±4.7/2.0±4.3	0.023
History of CAD, n (%)	3 (11)	1 (4)	0.380
History of DM, n (%)	1 (4)	1 (4)	0.910
History of smoking, n (%)	6 (21)	5 (24)	0.960
History of hypertension, n (%)	4 (14)	2 (8)	0.500
SBP (mmHg)	123±14	124±15	0.372
DBP (mmHg)	77±9	81±10	0.521
Heart rate (beats/min)	75±10	78±10	0.420
P wave duration (ms)	94.0±14.0	89.0±12.0	0.180

Legend: WPW - Wolff-Parkinson-White syndrome; DAVNP - atrioventricular nodal reentrant tachycardia; BMI - body mass index; CAD - coronary artery disease; DM - Diabetes mellitus; SBP - systolic blood pressure; DBP - diastolic blood pressure.

### Changes in ECG parameters after RF catheter ablation

Changes in ECG parameters of WPW patients and DAVNP patients after RF catheter ablation were presented in Table 2. ECG revealed that the LAV_max_ and LVMI increased noticeably in patients with WPW compared with the patients with DAVNP before ablation (LAV_max_: 40±9.49 vs. 34±7.18, *P* = 0.02; LVMI: 86.69±16.94 vs. 78.35±8.46, *P* = 0.03); after successful RF catheter ablation, there were still significant differences in the LAV_max_ and LVMI between WPW patients and DAVNP patients (LAV_max_: 39±8.94 vs. 33±6.74, *P* = 0.02; 87.35±14.08 vs. 77.91±8.48, *P* = 0.005). In addition, the levels of SRs, SRe and SRa in the two groups showed significant differences before ablation; SRs from the 4-chamber view (LA) in patients with WPW was lower than in patients with DAVNP (1.60±0.30 vs. 1.74±0.29, *P = *0.005); a higher level of SRe was observed in WPW patients in contrast with DAVNP patients from the 4-chamber view (LA/RA) (LA: −1.56±0.27 vs. −1.75±0.32, *P = *0.001; RA: −1.50±0.33 vs. −1.70±0.42, *P = *0.015, respectively); there were noticeable differences in SRa from the 4-chamber view (LA/RA) and the 3-chamber view between WPW patients and DAVNP patients (4-chamber view (LA): −1.54±0.29 vs. −1.82±0.27, *P* = 0.001; 4-chamber view (RA): −1.74±0.31 vs. −1.85±0.33, *P* = 0.045; 3-chamber view: −1.69±0.50 vs. −1.86±0.40, *P* = 0.002, respectively). However, no significant differences were found between WPW patients and DAVNP patients after ablation (all *P*>0.05).

**Table 2:** Echocardiographic parameters of WPW patients and DAVNP patients before and after radiofrequency catheter ablation.

	Before ablation			*P* value	After ablation			*P* value
	WPW patients(n = 28)	DAVNP patients(n = 24)			WPW patients(n = 28)	DAVNP patients(n = 24)		
**Echocardiography**								
LAV_max_ (mL/m^2^)	40±9.49	34±7.18		0.022	39±8.94	33±6.74		0.021
LAV_min_ (mL/m^2^)	17±5.25	15±3.61		0.542	16±5.25	15±3.31		0.341
RAV_max_ (mL/m^2^)	29±7.21	30±6.63		0.632	28±6.81	29±5.81		0.128
RAV_min_ (mL/m^2^)	15±4.84	14±2.62		0.773	14±4.55	14±2.29		0.196
**SRs (S^−1^)**								
4-chamber view (LA)	1.60±0.30	1.74±0.29		0.005	1.83±0.33	1.85±0.36		0.708
4-chamber view (RA)	1.68±0.48	1.75±0.38		0.336	1.80±0.34	1.93±0.37		0.107
2-chamber view	1.53±0.44	1.63±0.28		0.129	1.70±0.50	1.82±0.50		0.116
3-chamber view	1.57±0.31	1.64±0.28		0.191	1.64±0.37	1.56±0.35		0.105
**SRe (S^−1^)**								
4-chamber view (LA)	−1.56±0.27	−1.75±0.32		0.001	−1.75±0.27	−1.76±0.33		0.813
4-chamber view (RA)	−1.50±0.33	−1.70±0.42		0.015	−1.59±0.34	−1.60±0.28		0.932
2-chamber view	−1.65±0.37	−1.68±0.35		0.407	−1.59±0.46	−1.59±0.43		0.976
3-chamber view	−1.53±0.42	−1.61±0.38		0.106	−1.65±0.34	−1.61±0.35		0.534
**SRa (S** ^−**1**^ **)**								
4-chamber view (LA)	−1.54±0.29	−1.82±0.27		0.001	−1.74±0.31	−1.74±0.30		0.902
4-chamber view (RA)	−1.74±0.31	−1.85±0.33		0.045	−1.80±0.43	−1.93±0.34		0.117
2-chamber view	−1.84±0.46	−1.84±0.48		1.000	−1.80±0.45	−1.88±0.46		0.231
3-chamber view	−1.69±0.50	−1.86±0.40		0.002	−2.01±0.50	−1.91±0.50		0.288

Legend: WPW - Wolff-Parkinson-White syndrome; DAVNP - atrioventricular nodal reentrant tachycardia; LAV_max_ - left atrial maximum volume; LAV_min_ - left atrial minimal volume; RAV_max_ - right atrial maximum volume; RAV_min_ - right atrial minimal volume; SR - strain rate; SRs - systolic strain rate; SRe - early diastolic strain rate; SRa - late diastolic strain rate.

### Differences in ECG results in WPW patients with or without AF

Clinical and conventional ECG variables between the AF group and the non-AF group at baseline are presented in Table 3. Seven WPW patients (5 males and 2 females; mean age, 40±11.73) had ECG-confirmed AF before RFCA, and 21 WPW patients (12 males and 9 females; mean age, 40±15.83) showed no evidence of AF. There were no significant differences between clinical and conventional ECG variables between the AF group and the non-AF group (all *P*>0.05). Levels of SRs (1.40±0.21 vs. 1.60±0.26, *P* = 0.002), SRe (−1.37±0.26 vs. −1.80±0.41, *P* = 0.011), and SRa (−1.33±0.11 vs. −1.91±0.45, *P* = 0.009) from the 4-chamber view (LA) in the AF group before ablation differed significantly from recordings after ablation. The level of SRa from the 4-chamber view (RA) before ablation was also significantly higher than that after ablation (−1.72±0.29 vs. −1.98±0.30, *P* = 0.029). In the non-AF group, SRe levels decreased significantly after ablation in the 4-chamber view (LA/RA) (LA: −1.62±0.25 vs. −1.73±0.30, *P* = 0.028; RA: −1.54±0.32 vs. −1.74±0.41, *P* = 0.036, respectively); significant differences in SRa levels were also found in the 4-chamber view (LA) and 3-chamber view, both before and after ablation (4-chamber view (LA): −1.61±0.29 vs. −1.79±0.19, *P* = 0.018; 3-chamber view: −1.74±0.53 vs. −1.90±0.40, *P* = 0.019).

**Table 3:** Changes in echocardiographic variables before and after radiofrequency catheter ablation in the AF group and the non-AF group.

	AF group (n = 7)		*P* value	Non-AF group (n = 21)		*P* value
	Before ablation	After ablation		Before ablation	After ablation	
**Echocardiography**						
LAV_max_ (mL/m^2^)	23.81±5.31	21.60±4.90	0.102	23.32±4.72	22.91±4.06	0.318
LAV_min_ (mL/m^2^)	9.58±2.33	8.50±2.79	0.161	9.99±3.19	9.84±3.10	0.667
RAV_max_ (mL/m^2^)	17.33±5.05	15.56±2.86	0.394	17.01±3.50	16.89±3.22	0.788
RAV_min_ (mL/m^2^)	9.07±3.59	8.31±2.21	0.687	8.52±2.42	8.32±2.11	0.405
**SRs (S^−1^)**						
4-chamber view (LA)	1.40±0.21	1.60±0.26	0.002	1.67±0.30	1.78±0.29	0.069
4-chamber view (RA)	1.50±0.31	1.74±0.33	0.092	1.75±0.52	1.76±0.40	0.909
2-chamber view	1.45±0.36	1.44±0.23	0.873	1.56±0.47	1.69±0.39	0.108
3-chamber view	1.41±0.20	1.51±0.17	0.337	1.62±0.33	1.68±0.30	0.346
**SRe (S^−1^)**						
4-chamber view (LA)	−1.37±0.26	−1.80±0.41	0.011	−1.62±0.25	−1.73±0.30	0.028
4-chamber view (RA)	−1.41±0.38	−1.56±0.43	0.265	−1.54±0.32	−1.74±0.41	0.036
2-chamber view	−1.51±0.21	−1.63±0.28	0.284	−1.69±0.40	−1.69±0.37	0.955
3-chamber view	−1.31±0.18	−1.38±0.35	0.571	−1.60±0.45	−1.68±0.36	0.112
**SRa (S^−1^)**						
4-chamber view (LA)	−1.33±0.11	−1.91±0.45	0.009	−1.61±0.29	−1.79±0.19	0.018
4-chamber view (RA)	−1.72±0.29	−1.98±0.30	0.029	−1.75±0.32	−1.81±0.34	0.337
2-chamber view	−1.89±0.75	−1.92±0.67	0.912	−1.82±0.34	−1.81±0.42	0.832
3-chamber view	−1.52±0.38	−1.74±0.38	0.074	−1.74±0.53	−1.90±0.40	0.019

Legend: AF - atrial fibrillation; LAV_max_ - left atrial maximum volume; LAV_min_ - left atrial minimal volume; RAV_max_ - right atrial maximum volume; RAV_min_ - right atrial minimal volume; SR - strain rate; SRs - systolic strain rate; SRe - early diastolic strain rate; SRa - late diastolic strain rate.

## Discussion

The present study results show that the LAV_max_ index, LV mass index, and average peak systolic SRs in patients with WPW syndrome were significantly higher than in patients with DAVNP, revealing that the AAV pathway may have a close association with atrial function impairment WPW syndrome patients. Although the exact mechanism through which AAV pathways contribute to impaired atrial function remain largely unidentified, we hypothesized that the critical determinants of impaired atrial function in WPW syndrome, which is suspected to play a vital role in AF, are the anterograde conduction properties of [27]. AP is the outcome of an embryological fault in the formation of fibrous tissue separating the atria and the ventricles, the abnormalities of which may be connected to the functional electrical properties of the atrium close to the AP [28]. Therefore, the existence of a functional AP is believed to play an important role in promoting atrial vulnerability and triggering AF in WPW syndrome patients [29], [30]. This hypothesis is consistent with a previous study that has demonstrated that the anterograde conduction properties, in addition to the presence of the AP, may play an important role in the spontaneous occurrence of impaired atrial function [31]. In addition, results show that the average peak systolic SRs in patients with WPW syndrome were significant higher in those in patients with dominant bypass than those patients with recessive bypass. It can be interpreted that this result indicates a significant improvement in the atrial tissue function, thus suggesting a more serious bypass-related atrial vulnerability, and recessive bypass WPW patients only have partial functional recovery, revealing a higher proportion of inherent atrial vulnerability.

The results of our study also show that the atrial function of WPW syndrome patients was significantly improved after RF catheter ablation, suggesting that RF catheter ablation might significantly improve local atrial reservoir, conduit, and booster pump function in patients with WPW. However, the specific mechanism by which RF catheter ablation would significantly affect atrial function in WPW syndrome patients remains only partially understood. It is supposed that RF catheter ablation might interrupt the normal AAV pathway conduction system for palliation of AF or flutter, eliminating conduction through atrioventricular accessory pathways, modify atrioventricular nodal conduction for control of AVNRT, and ablate the site of origin of primary atrial tachycardias [32]. Our results are in accordance with a previous study reporting that RF catheter ablation can result in maintenance of normal atrial function in a majority of patients with WPW syndrome [33]. However, there was also a possibility of the atrial vulnerability extending after RF catheter ablation; with this in mind, the usage of two-dimensional speckle tracking echocardiography imaging in WPW patients in a long-term follow-up period was necessary in the evaluation and improvement of the overall function of RF catheter ablation. Generally speaking, speckle tracking echocardiography, as an echocardiographic imaging technique, analyzes the motion of tissues in the heart by using ultrasonic sound waves for the purpose of generating interference patterns and natural acoustic reflections. With tissue deformation and motion, the two-dimensional strained-based sequences do provide both quantitative and qualitative information [34]. Most importantly, two-dimensional speckle tracking echocardiography overcomes a series limitations, such as angle dependency, noise interference, and substantial variability between intra-observer and inter-observer, which other speckle tracking technologies, such as tissue Doppler technology, face [35]–[37].

Our findings also indicate that patients with WPW syndrome and concomitant AF have worse atrial function than WPW patients without AF, revealing that AF may be significantly associated with atrial function. Although the exact association of this complication with atrial function in the WPW population also remains unclear, the potential causes may involve frequent tachycardias promoting an electrical remodeling and increased atrial vulnerability to AF [29], [38]. It should be noted that the appearance of AF in patients with WPW syndrome may result in atrial function impairment, which seems to be due to severe hypotension associated with fast ventricular response and degeneration of the rhythm into ventricular fibrillation [14], [15]. Our results are in better agreement with the fossil record and suggest that WPW increases vulnerability of the atrium due to the presence of AF, which may play a significant functional role in atrioventricular reentrant tachycardia [21].

There are several methodological limitations associated with this retrospective study that warrant consideration. Firstly, we failed to identify AF during intraoperative electrophysiological examination, which led to the unrecognized exclusion of the potential AF patients in the AF group; this may affect the comparison of differences between the two groups. Secondly, two-dimensional STE technique is frame-dependent, needs high quality grey-scale images, and requires a learning curve for the off-line analysis; this might limit further assessment of AF and vulnerability in WPW patients [39]. Additionally, further understanding of the exact mechanism of AF in WPW patients requires combining electrophysiologic variables and atrial function; this was not included in the present study [40]. Caution is also warranted in interpreting these findings because of the short duration of the follow-up and small sample size, and sufficient power was still needed to observe the recovery of atrial function and incidence of AF after ablation of AP.

Our findings support the hypothesis that the existence of a functional AP is critical in the increased development of atrial vulnerability, which triggers AF in the WPW syndrome. Additionally, the RF catheter ablation of AAV pathway can effectively improve atrial function in WPW patients. Finally, the usage of two-dimensional speckle tracking echocardiography imaging in WPW patients in a long-term follow-up period function helps significantly in patient evaluation and improves the overall function of RF catheter ablation. However, due to the limitations mentioned above, further detailed studies are still required to confirm our findings.
